# Association of blood lipids with coronary artery plaque among Saudi patients referred to computed tomography

**DOI:** 10.1186/s12872-022-02690-x

**Published:** 2022-06-03

**Authors:** Sumaya Al Helali, Muhammad Abid Hanif, Ahmad Al Majed, Nura Alshugair, Abdullah Belfageih, Hamad Al Qahtani, Sameer Al Dulikan

**Affiliations:** Adult Cardiology Department, Prince Sultan Cardiac Centre, Riyadh, 12233 Saudi Arabia

**Keywords:** Computed tomography, Angiography, Plaques, Atherosclerosis, Gender, Saudi Arabia

## Abstract

**Background:**

Blood lipids are strong risk factors for the progression of atherosclerotic plaques. However, data on gender-specific associations are limited.

**Objectives:**

To examine gender-specific associations of coronary plaque with blood lipids among a large sample of Saudi patients without CAD.

**Methods:**

Retrospective cross-sectional study was conducted among adult patients referred to (64 multidetector spiral) computed tomography (CT) for standard indications at the Prince Sultan Cardiac Centre (Riyadh, Saudi Arabia) between July 2007 and December 2017. Those with pre-existing CAD were excluded. Plaques were determined based on quantification of coronary calcium and Coronary CT angiography.

**Results:**

A total 2421 patients (1498 males and 923 females) were included. The prevalence of any plaque was 36.6% with higher burden in males than females (41.3% versus 28.9%, *p* < 0.001). Approximately 78.9% of all plaques were calcified. Blood lipids (mmol/L) were 4.75 ± 1.14 for total cholesterol, 2.90 ± 0.96 for LDL cholesterol, 1.20 ± 0.36 for HDL cholesterol, and 1.64 ± 1.09 for triglycerides. Males had significantly higher triglycerides and lower HDL cholesterol compared with females. In adjusted models in males and all patients, soft and/or calcified plaques were significantly associated with lower HDL cholesterol and higher triglycerides. In females, the only significant association was between soft plaques and higher triglycerides.

**Conclusions:**

Middle-aged patients without clinical CAD in Saudi Arabia have a high burden of plaques, specially calcified ones. The findings may impact the use of lipid lowering mediations, by underscoring the importance of assessing the risk of CAD in patients without clinical CAD even in case of lack of coronary calcification.

## Introduction

Atherosclerosis is a chronic inflammatory disease characterized by build‑up of fatty plaques in the walls of arteries, eventually leading to their narrowing [1]. Rupture of coronary atherosclerotic plaque can lead to thrombosis with subsequent coronary artery disease (CAD) events and mortality [2, 3]. Globally, CAD is the leading cause of death in all countries, irrespective of their income groups [4]. Similarly, in Saudi Arabia, CAD is the leading cause of deaths with approximately 114 deaths per 100,1000 population annually [5]. This represents approximately 17% of all hospital deaths [6]. Additionally, cardiovascular disease is the major cause of disability among Saudi adults as measured by disability-adjusted life-years [7].

Hyperlipidemia particularly high levels of low-density lipoprotein (LDL) cholesterol is one of the leading risk factors of CAD and stroke [8, 9]. Over the last decades, the prevalence and burden of hyperlipidemia is increasing, specially in developing countries, due to widespread adoption of unhealthy lifestyle and increased prevalence of diabetes and obesity [10, 11]. The pattern in Middle Eastern countries is peculiar and largely manifested as low levels of high-density lipoprotein (HDL) cholesterol and high levels of triglycerides [9, 10]. On the other hand, the use of statins specially in developed countries significantly reduced the average plasma cholesterol levels and hyperlipidemia-associated mortality [9, 12].

The prevalence of hyperlipidemia in Saudi Arabia has been estimated between 20 and 50% in the general population [13–15]. However, gender-specific levels of blood lipids among patients suspected of atherosclerosis but without history of CAD has not been comprehensively examined. Additionally, the data on the prevalence of plaques and their association with blood lipids are limited [16]. The objective of the current study was to examine gender-specific associations of blood lipids with coronary plaque among in a large sample of Saudi patients without clinical CAD.

## Methods

### Setting

The current study was conducted at Prince Sultan Cardiac Center (PSCC). The PSCC is 200-bed specialized cardiac center located in Riyadh that provides a major portion of the diagnostic and therapeutic cardiac services in Saudi Arabia. The PSCC has several departments including adult and pediatric cardiology, adult and pediatric cardiac surgery, cardiac anaesthesia, and advanced imaging. The current study was done at the advanced imaging unit under adult cardiology.

### Design

It was a retrospective cohort study conducted between July 2007 and December 2017. The study design obtained all required ethical approvals from the ethical committee of PSCC.

### Population

The study targeted adult patients (age > 18 years) referred to (64 multidetector spiral) computed tomographic (CT) for standard indications. Those with pre-existing CAD were excluded from the study. Pre-existing CAD was defined as myocardial infarction, angioplasty, stent placement, and coronary artery bypass grafting. Additionally, CT done for aortic assessment, for pericardial assessment and low-quality CT with artifacts were excluded from the study. Finally, those who were missing blood lipid (N = 441) or plaque (n = 20) testing results were excluded, leaving 2421 for analysis. The number of patients is sufficient to detect a gender-specific difference in total cholesterol of 0.15 mmol/L with more than 90% power.

### CT scanning protocol

Patients were scanned during a single breath-hold using a 64 (multidetector spiral) CT scanner (Philips Brilliance). A retrospective gating protocol with thickness of 0.5 to 2.5, FOV 220, and the average radiation dose is 6–9 mSv. The scanning protocol was designed to minimize the radiation dose based on BMI. Indications of coronary CT included chest pain in patients with intermediate risk of CAD, impaired left ventricular function in asymptomatic patient, before non-coronary cardiac surgery in patients with intermediate risk of CAD, to rule out coronary anomaly, and in case of arrhythmia with atypical chest pain.

### Definitions

**Fig. 1:**
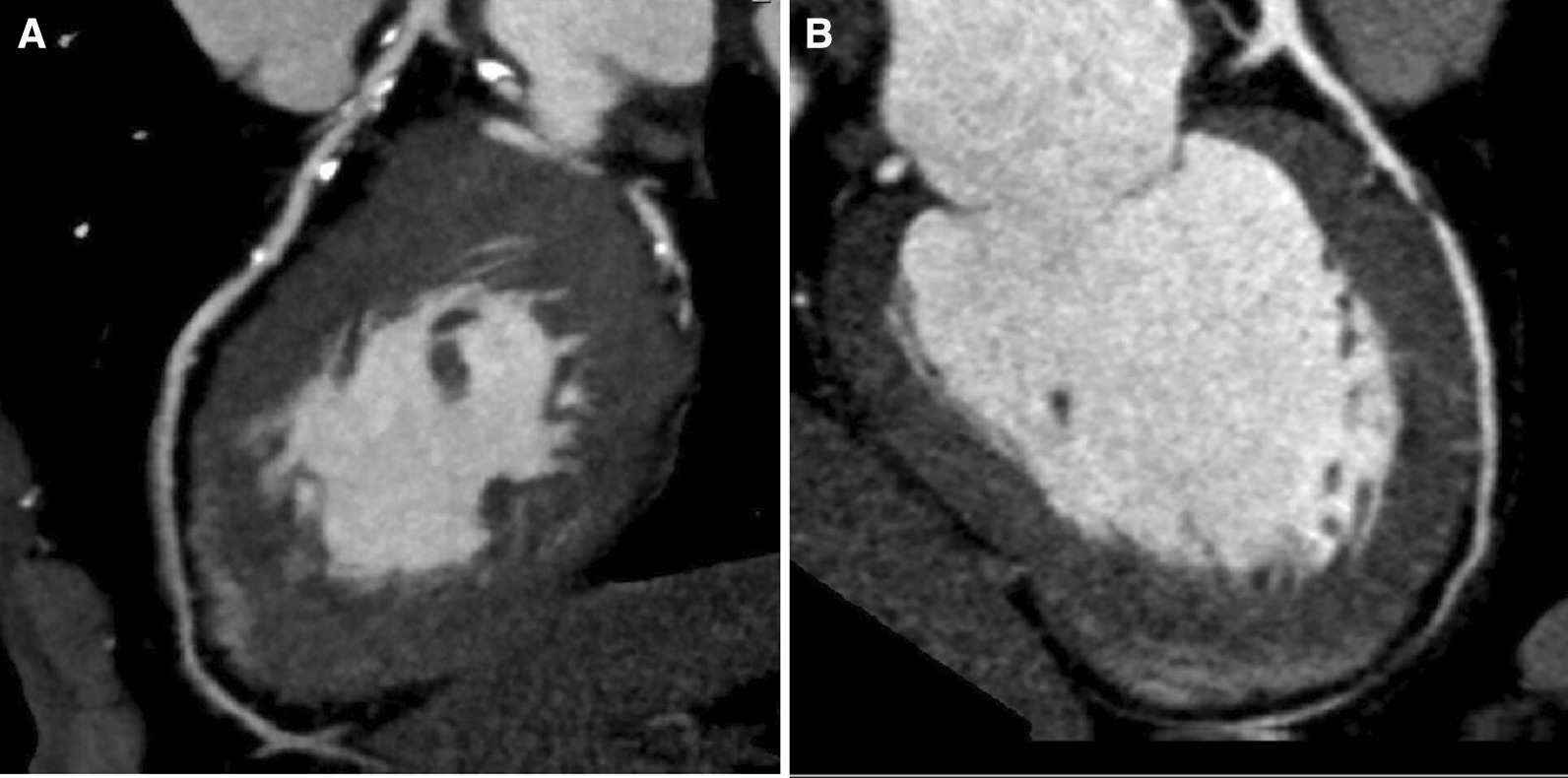
A patient with clacified plaque (**A**). Coronary CT angiography showed proximal LAD calcified lesion in a patient with total coronary calcium score 86 HU. A patient with soft (non-calcified) plaque (**B**). Coronary CT angiography showed proximal LAD non-cacified lesion in a patient with total coronary calcium score zero

Plaques were determined based on quantification of coronary calcium and coronary CT angiography. Classification of plaques as calcified or soft (non-calcified) was based on the presence or lack of coronary calcification (respectively). The later was expressed using Agatston score, which reflects the total area of calcium deposits and the density of the calcium in the entire coronary tree. Therefore, those with coronary stenosis on coronary CT angiography and had above zero coronary calcium score were classified as calcified plaque (Fig. 1A) while those with coronary stenosis on coronary CT angiography and had zero coronary calcium score were classified as soft plaque (Fig. 1B). Patients were classified as no plaques, soft plaques, and calcified plaques. Blood lipids results at the time of the computed tomography were used. The levels defining controlled blood lipids (in mmol/L) were < 5.17 for total cholesterol, ≥ 1.0 for males and ≥ 1.3 in females for HDL cholesterol, and < 1.7 for triglycerides. The levels defining control of LDL cholesterol (in mmol/L) was < 3 in mild SCORE risk, < 2.6 in moderate SCORE risk, < 1.8 in high SCORE risk, and < 1.4 in very high SCORE risk.

### Risk stratification

Stratification of the risk of CAD among patients was done using the number of risk factors and the systematic coronary risk evaluation (SCORE) of the European Society of Cardiology (ESC). Risk factors for CAD were defined as history of hypertension, diabetes, dyslipidemia, smoking, family history of premature CAD (before the age of 65 years), and obesity (BMI > 30). The SCORE estimating the 10-year risk of developing fatal cardiovascular disease in populations with high cardiovascular disease risk was calculated according to standard methodology [17].

### Data collection tool

Study data collection sheet was initiated for patients who underwent coronary CT and meeting the study eligibility criteria. Clinical information including medical history, traditional risk factors, and cardiac comorbidity were then abstracted from the electronic patient chart system.

### Statistical analysis

Demographic characteristics, risk stratification, and blood lipids were compared by the plaque and gender groups. Chi-square or Fisher exact tests (as appropriate) were used to detect differences in categorical variables. Analysis of variance (ANOVA) or Kruskal–Wallis test (as appropriate) were used to detect plaque differences in continuous variables while t-test or Mann–Whitney test (as appropriate) were used to detect gender differences in continuous variables. In case of plaque groups, pairwise differences were calculated using Bonferroni adjustment method for multiple comparisons. Generalized linear regression models was used to detect differences in log-transformed blood lipid levels between plaque and gender groups after adjusting for other CAD risk factors. Log data were transformed back to normal before presentation in Table 4. Plaque-gender interaction terms were included in the models that were not split by gender to test for the significance of interaction between plaque and gender. All P-values were two-tailed. *P*-value < 0.05 was considered as significant. SPSS software (release 25.0, Armonk, NY: IBM Corp) was used for all statistical analyses.

## Results

**Fig. 2:**
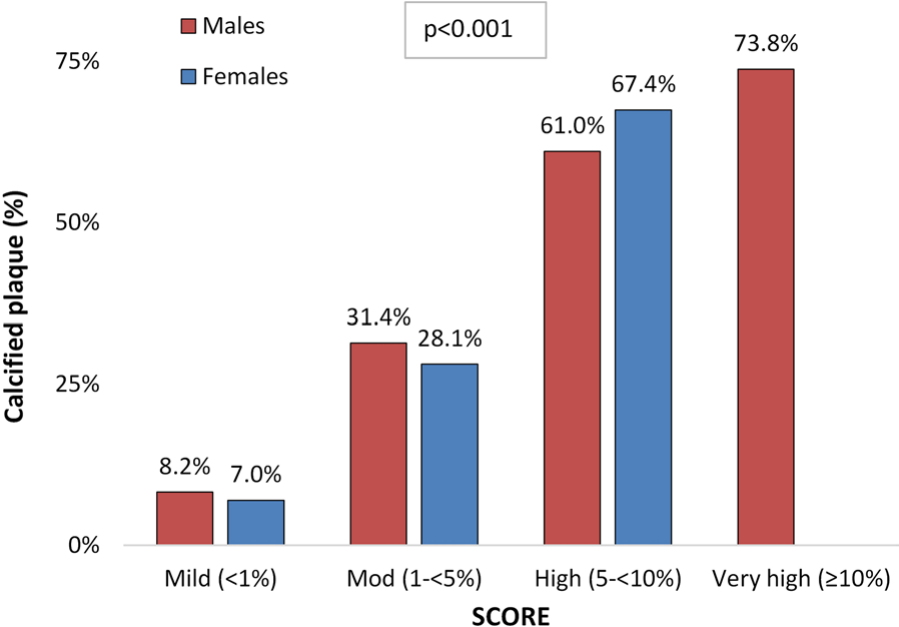
Frequency of coronary plaque by gender

A total 2421 patients (1498 males and 923 females) were included in the current analysis. As shown in Fig. 2, the prevalence of any plaque was 36.6% and was higher in males than females (41.3% versus 28.9%, *p* < 0.001). Approximately 78.9% of all plaques were calcified. The prevalence of calcified plaques was 28.9% and was higher in males than females (33.0% versus 22.1%, *p* < 0.001). The prevalence of soft plaques was 7.7% and was higher in males than females (8.3% versus 6.8%, *p* = 0.018).

Table 1 shows the demographic and clinical characteristics by plaque status. The majority (61.9%) were males. The average age was 50.3 ± 11.5 years and the average body mass index (BMI) was 30.2 ± 5.6. The average systolic blood pressure was 129.2 ± 20.3 mmHg. The most common CAD risk factors included obesity (45.9%), hypertension (39.2%), diabetes (28.6%), hyperlipidemia (17.1%), current smoking (12.3%), and family history of premature cad (8.1%). According to the SCORE, 10-year risk of developing fatal cardiovascular disease was mild (< 1% risk) in 23.0% of the patients, moderate (1 to < 5% risk) in 62.8%, high (5 to < 10% risk) in 11.5%, and very high (≥ 10% risk) in 2.7%. Adding diabetes to the SCORE increased the percentages of high and very high risk to 35.7% and 6.9%, respectively. Calcified and/or soft plaques were significantly associated with older age, male gender, obesity, hypertension, diabetes, hyperlipidemia, current smoking, renal impairment, higher SCORE, and higher levels of fasting blood glucose, hemoglobin A1c, and serum creatinine.

**Table 1:** Demographic and clinical characteristics of the patients by coronary plaque groups

	No plaque	Soft plaque	Calcified plaque	Total	*p*-value	Test
**Age**						
Mean ± SD	46.7 ± 10.5	50.4 ± 10.4	58.1 ± 9.9	50.3 ± 11.5	< 0.001	ANOVA
< 45	627 (40.8%)	49 (26.2%)	57 (8.2%)	733 (30.3%)	< 0.001	Chi
45–64	847 (55.2%)	121 (64.7%)	457 (65.4%)	1425 (58.9%)		
≥ 65	61 (4.0%)	17 (9.1%)	185 (26.5%)	263 (10.9%)		
**Sex**						
Male	879 (57.3%)	124 (66.3%)	495 (70.8%)	1498 (61.9%)	< 0.001	Chi
Female	656 (42.7%)	63 (33.7%)	204 (29.2%)	923 (38.1%)		
**Body mass index**						
Mean ± SD	30.1 ± 5.7	30.8 ± 5.8	30.1 ± 5.3	30.2 ± 5.6	0.254	ANOVA
Normal	281 (19.0%)	17 (9.2%)	107 (15.5%)	405 (17.2%)	0.006	Chi
Overweight	522 (35.2%)	75 (40.8%)	272 (39.4%)	869 (36.9%)		
Obese	679 (45.8%)	92 (50.0%)	312 (45.2%)	1083 (45.9%)		
**Blood pressure (mmHg)**						
Systolic	127.1 ± 17.6	133.6 ± 19.2	132.6 ± 25.0	129.2 ± 20.3	< 0.001	ANOVA
Diastolic	72.5 ± 12.0	72.8 ± 11.2	73.5 ± 12.7	72.8 ± 12.2	0.209	ANOVA
Fasting glucose (mmol/L)	6.05 ± 2.42	7.75 ± 3.65	6.67 ± 3.67	6.36 ± 2.98	< 0.001	KW
Hemoglobin A1c	6.4 ± 1.5	7.3 ± 2.1	6.7 ± 1.8	6.5 ± 1.7	< 0.001	KW
Creatinine (mmol/L)	73.8 ± 35.2	81.6 ± 96.1	78.0 ± 55.9	75.6 ± 49.0	0.003	KW
Chronic kidney disease*						
Normal (> 90)	970 (63.6%)	117 (62.9%)	378 (54.2%)	1465 (60.8%)	< 0.001	Chi
Mild (60–89)	523 (34.3%)	64 (34.4%)	279 (40.0%)	866 (35.9%)		
Moderate (30–59)	27 (1.8%)	3 (1.6%)	33 (4.7%)	63 (2.6%)		
Severe (15–29)	1 (0.1%)	0 (0.0%)	5 (0.7%)	6 (0.2%)		
ESRD (< 15)	4 (0.3%)	2 (1.1%)	3 (0.4%)	9 (0.4%)		
**Risk factors**						
Diabetes	334 (21.8%)	85 (45.5%)	274 (39.2%)	693 (28.6%)	< 0.001	Chi
Hypertension	495 (32.3%)	90 (48.1%)	363 (51.9%)	948 (39.2%)	< 0.001	Chi
Hyperlipidemia	193 (12.6%)	53 (28.3%)	166 (23.9%)	412 (17.1%)	< 0.001	Chi
Current smoking	158 (10.3%)	43 (23.0%)	97 (13.9%)	298 (12.3%)	< 0.001	Chi
Family history of premature CAD	116 (7.6%)	14 (7.5%)	66 (9.4%)	196 (8.1%)	0.309	Chi
**SCORE without diabetes**						
Mild risk (< 1%)	447 (32.3%)	26 (14.7%)	38 (5.7%)	511 (23.0%)	< 0.001	Chi
Moderate (1 to < 5%)	853 (61.6%)	123 (69.5%)	422 (63.6%)	1398 (62.8%)		
High risk (5 to < 10%)	75 (5.4%)	22 (12.4%)	159 (23.9%)	256 (11.5%)		
Very high risk (≥ 10%)	10 (0.7%)	6 (3.4%)	45 (6.8%)	61 (2.7%)		
**SCORE with diabetes**						
Mild risk (< 1%)	358 (25.6%)	15 (8.3%)	24 (3.5%)	397 (17.6%)	< 0.001	Chi
Moderate (1 to < 5%)	604 (43.1%)	62 (34.3%)	234 (34.5%)	900 (39.8%)		
High risk (5 to < 10%)	391 (27.9%)	82 (45.3%)	333 (49.1%)	806 (35.7%)		
Very high risk (≥ 10%)	48 (3.4%)	22 (12.2%)	87 (12.8%)	157 (6.9%)		

ESRD, end stage renal disease; CAD, coronary artery disease; SCORE, Systematic Coronary Risk Estimation of ESC/EAS; FRS, Framingham Risk Score; ANOVA, analysis of variance test; Chi. Chi-square test; KW, Kruskal–Wallis test

*Using glomerular filtration rate in mL/min

Table 2 shows the crude means and standard deviations of blood lipids by plaque and gender groups. Overall, the levels of blood lipids (mmol/L) were 4.75 ± 1.14 for total cholesterol, 2.90 ± 0.96 for LDL cholesterol, 1.20 ± 0.36 for HDL cholesterol, and 1.64 ± 1.09 for triglycerides. Plaques were significantly associated with all blood lipids. Soft and calcified plaques were significantly associated with higher triglycerides and lower HDL cholesterol, with worse profile in soft than calcified plaques. Additionally, calcified plaques were significantly associated with lower total and LDL cholesterol. Males had significantly higher triglycerides and lower HDL cholesterol compared with females. In males only, the associations between plaques and blood lipids were almost identical to overall associations, with the exception of the association between calcified plaques and higher triglycerides that did not reach statistical significance. In females only, soft plaques were significantly associated with lower HDL cholesterol while calcified plaques were significantly associated with higher triglycerides.

**Table 2:** Crude means and standard deviations of blood lipids (mmol/L) by coronary plaque and gender groups

	No plaque	Soft plaque	Calcified plaque	Total	*P*-value^1^	*P*-value^2^	Pairwise difference
**Males**							
Total cholesterol	4.81 ± 1.06	4.76 ± 1.27	4.58 ± 1.23	4.73 ± 1.14	< 0.001	0.328	B
LDL cholesterol	3.01 ± 0.89	2.86 ± 1.04	2.77 ± 1.04	2.92 ± 0.96	< 0.001	0.151	B
HDL cholesterol	1.15 ± 0.31	1.01 ± 0.28	1.10 ± 0.30	1.12 ± 0.30	< 0.001	< 0.001	A, B, C
Triglycerides	1.65 ± 1.03	2.12 ± 1.86	1.73 ± 1.11	1.72 ± 1.16	0.006	< 0.001	A, C
**Females**							
Total cholesterol	4.81 ± 1.13	4.75 ± 1.28	4.75 ± 1.08	4.79 ± 1.13	0.610	0.328	
LDL cholesterol	2.90 ± 0.96	2.83 ± 0.97	2.76 ± 0.90	2.86 ± 0.95	0.110	0.151	
HDL cholesterol	1.33 ± 0.40	1.23 ± 0.41	1.33 ± 0.40	1.32 ± 0.40	0.036	< 0.001	A
Triglycerides	1.46 ± 0.87	1.73 ± 1.57	1.65 ± 1.01	1.52 ± 0.97	0.012	< 0.001	B
**Total**							
Total cholesterol	4.81 ± 1.09	4.76 ± 1.27	4.63 ± 1.19	4.75 ± 1.14	< 0.001		B
LDL cholesterol	2.96 ± 0.92	2.85 ± 1.01	2.76 ± 1.00	2.90 ± 0.96	< 0.001		B
HDL cholesterol	1.22 ± 0.36	1.09 ± 0.34	1.17 ± 0.35	1.20 ± 0.36	< 0.001		A, B, C
Triglycerides	1.57 ± 0.97	1.99 ± 1.77	1.70 ± 1.08	1.64 ± 1.09	< 0.001		A, B, C

*P*-value^1^ indicates differences between plaque groups using Kruskal–Wallis test; *P*-value^2^ indicates differences between males and females using Mann–Whitney test; pairwise difference indicates significant differences using Bonferroni adjustment method for multiple comparisons between (A) no plaque versus soft plaque, (B) no plaque versus calcified plaque, and (C) soft plaque versus calcified plaque. LDL, low-density lipoprotein; HDL, high-density lipoprotein

Table 3 shows the control of blood lipids by plaque and gender groups. The control of blood lipids was 66.9% for total cholesterol, 66.9% for triglycerides, 59.3% for HDL cholesterol, and 29.2% for LDL cholesterol. Males had significantly better control of HDL cholesterol but worse control of LDL cholesterol and triglycerides compared with females. Soft and calcified plaques were significantly associated with lower control of HDL cholesterol in males and all patients. Soft plaques were significantly associated with lower control of triglycerides in all patients only. Calcified plaques were significantly associated with higher control of total cholesterol in males only. Soft and calcified plaques were not significantly associated with control of blood lipids in females.

**Table 3:** Control of blood lipids by coronary plaque and gender groups

	No plaque	Soft plaque	Calcified plaque	Total	*P*-value^1^	*P*-value^2^	Pairwise difference
**Males**							
Total cholesterol	561 (64.0%)	78 (62.9%)	353 (71.3%)	992 (66.3%)	0.015	0.453	B
LDL cholesterol	216 (27.9%)	30 (25.0%)	126 (26.3%)	372 (27.1%)	0.721	0.005	
HDL cholesterol	602 (70.7%)	65 (52.4%)	297 (60.1%)	964 (65.6%)	< 0.001	< 0.001	A, B
Triglycerides	570 (65.0%)	69 (55.6%)	315 (63.9%)	954 (63.9%)	0.128	< 0.001	
**Females**							
Total cholesterol	440 (67.2%)	46 (73.0%)	139 (68.1%)	625 (67.8%)	0.634	0.453	
LDL cholesterol	219 (35.0%)	18 (29.5%)	52 (26.1%)	289 (32.6%)	0.059	0.005	
HDL cholesterol	321 (50.6%)	24 (38.1%)	98 (48.0%)	443 (49.1%)	0.159	< 0.001	
Triglycerides	482 (73.7%)	42 (66.7%)	138 (67.6%)	662 (71.9%)	0.155	< 0.001	
**Total**							
Total cholesterol	1001 (65.3%)	124 (66.3%)	492 (70.4%)	1617 (66.9%)	0.062		
LDL cholesterol	435 (31.0%)	48 (26.5%)	178 (26.3%)	661 (29.2%)	0.055		
HDL cholesterol	923 (62.1%)	89 (47.6%)	395 (56.6%)	1407 (59.3%)	< 0.001		A, B
Triglycerides	1052 (68.7%)	111 (59.4%)	453 (65.0%)	1616 (66.9%)	0.016		A

*P*-value^1^ indicates differences between plaque groups using chi-square; *P*-value^2^ indicates differences between males and females using chi-square; pairwise difference indicates significant differences using chi-square for multiple comparisons between (A) no plaque versus soft plaque, (B) no plaque versus calcified plaque, and (C) soft plaque versus calcified plaque. LDL, low-density lipoprotein; HDL, high-density lipoprotein

Table 4 shows adjusted means and standard errors of blood lipids by plaque and gender groups. Generalized linear regression models adjusted for other potential plaque risk factors (listed in Table 4 notes) showed that males had significantly higher triglycerides and lower HDL/total cholesterol compared with females. In males and all patients, soft and/or calcified plaques were significantly associated with lower HDL cholesterol and higher triglycerides. In females, the only significant association was between soft plaques and higher triglycerides. Plaque-gender interactions were significant in HDL cholesterol and triglycerides models and marginally significant in total cholesterol model.

**Table 4:** Adjusted* means and standard errors of blood lipids (mmol/L) by coronary plaque and gender groups

	No plaque	Soft plaque	Calcified plaque	Total	*P*-value^1^	*P*-value^2^	Pairwise difference
**Males**							
Total cholesterol	4.66 ± 1.02	4.60 ± 1.03	4.56 ± 1.02	4.60 ± 1.01	0.457	0.005	
LDL cholesterol	2.81 ± 1.02	2.66 ± 1.04	2.70 ± 1.02	2.73 ± 1.02	0.169	0.730	
HDL cholesterol	1.10 ± 1.02	0.98 ± 1.03	1.03 ± 1.02	1.05 ± 1.01	< 0.001	< 0.001	A, B
Triglycerides	1.55 ± 1.03	1.76 ± 1.06	1.65 ± 1.04	1.60 ± 1.03	0.030	< 0.001	A
**Females**							
Total cholesterol	4.62 ± 1.03	4.68 ± 1.04	4.81 ± 1.03	4.76 ± 1.01	0.185	0.005	
LDL cholesterol	2.65 ± 1.05	2.67 ± 1.07	2.70 ± 1.05	2.75 ± 1.02	0.859	0.730	
HDL cholesterol	1.25 ± 1.04	1.17 ± 1.05	1.28 ± 1.04	1.25 ± 1.02	0.128	< 0.001	
Triglycerides	1.21 ± 1.07	1.31 ± 1.10	1.37 ± 1.07	1.39 ± 1.03	0.024	< 0.001	B
**Total**							
Total cholesterol	4.66 ± 1.01	4.63 ± 1.02	4.64 ± 1.01	4.64 ± 1.01	0.921	0.072	
LDL cholesterol	2.76 ± 1.02	2.69 ± 1.03	2.72 ± 1.02	2.72 ± 1.02	0.476	0.367	
HDL cholesterol	1.15 ± 1.02	1.02 ± 1.03	1.07 ± 1.02	1.08 ± 1.02	< 0.001	< 0.001	A, B
Triglycerides	1.45 ± 1.03	1.66 ± 1.05	1.62 ± 1.03	1.57 ± 1.03	< 0.001	< 0.001	A, B

*P*-value^1^ indicates differences between plaque groups using F test; *P*-value.^2^ indicates differences between males and females using F test; the *p*-values shown in the “total” group represent the significance of plaque-gender interaction terms; pairwise difference indicates significant differences using Bonferroni adjustment method for multiple comparisons between (A) no plaque versus soft plaque, (B) no plaque versus calcified plaque, and (C) soft plaque versus calcified plaque. LDL, low-density lipoprotein; HDL, high-density lipoprotein

*Adjusted for age, body mass index, fasting blood glucose, hemoglobin A1c, systolic blood pressure, serum creatinine, and history of cardiovascular risk factors including hypertension, diabetes, smoking, and family history of premature coronary artery disease

## Discussion

The current study reported gender-specific prevalence of plaques, levels of blood lipids, and their associations among patients without history of CAD in Saudi Arabia. Approximately 37% of the patients in the current study had plaques, with 79% of them had calcified plaques. The current finding confirms the high burden subclinical atherosclerosis among middle-aged patients without clinical CAD [18]. Additionally, it reconfirm the role of coronary CT-angiography as an important screening tool for subclinical atherosclerosis even in case of lack of coronary calcification [3]. As expected, the current data showed higher risk of plaques specially calcified ones in males compared with females [19]. The prevalence of plaques in the current study was probably lower than reported in several international studies [20–23]. However, the plaques had probably higher percentage of calcification, indicating advanced progression of atherosclerosis in this relatively young age groups [20–23]. For example, plaques in previous studies were reported in 60% of patients without CAD with mixed levels of CAD risk [21, 22] and 20% in patients without CAD with low CAD risk [23]. In these studies, non-calcified plaques represented between 50 and 75% of all plaques [20–23]. Comparing the current findings with local studies is challenging due to limited data and different methodology. Nevertheless, soft plaques in Saudi Arabia were found in 6.4% of symptomatic patients with a high clinical suspicion of CAD but without coronary calcification [16].

The levels of blood lipids in the current study were slightly different from global levels [9]. For example, total cholesterol levels were slightly higher than global levels (4.73 versus 4.66 in males and 4.79 vs. 4.75 in females) while HDL cholesterol levels were very similar to the global levels (1.12 vs. 1.12 in males and 1.32 vs. 1.29 in females) [9]. On the other hand, the frequency of hyperlipidemia in the current study was very similar to those reported in the Middle Eastern and Gulf countries (33.1% vs. 32.7% to 36.8%) [10, 11]. Males in the current study had higher triglycerides and lower HDL cholesterol compared with females. This was observed in previous reports in Saudi Arabia, using both national population data [13, 14] and outpatient clinic data [24]. Adjustment for traditional CAD risk factors in the current study attenuated the values of all blood lipids but did not change the gender-specific differences in triglycerides and HDL cholesterol. Males in the current study had better control of HDL cholesterol but worse control of triglycerides compared with females. Interestingly, recent guidelines recommend the use of lipid lowering medications in both males and females according to the patients' risk of CAD, rather than treating to specific lipid levels [25, 26].

Similar to previous studies, coronary plaques in the current study were associated with traditional risk factors of CAD including the SCORE [21, 27, 28]. While the patients in the current study had no history of clinical CAD, they had mixed risk of CAD, which was largely of mild/intermediate degree. The associations between plaques and blood lipids in the current study were stronger in males than females in both univariate and multivariate analysis. For example, males who had lower HDL cholesterol and higher triglycerides than females had stronger independent associations between soft and/or calcified plaques and lower HDL cholesterol and higher triglycerides. The gender-specific differences in the associations of coronary plaque with blood lipids may be related to the sex-hormone associated delayed atherosclerotic pathology including lipid accumulation [29, 30]. HDL cholesterol has been shown to inhibit the progression of atherosclerotic plaques by moving cholesterol from macrophages in the arterial wall of the arteries to the liver [31]. Higher triglycerides and lower HDL cholesterol were recognized as atherogenic index, which is associated with progression of atherosclerotic plaques [32]. Additionally, it is associated with other conditions that promote atherosclerosis such as insulin resistance and metabolic syndrome [32]. It should be noted that most of circulating triglycerides are carried on triglycerides-rich very low-density lipoprotein (VLDL). The later are directly involved in fatty deposition on the arterial wall [33].

The current study is considered the first study in Saudi Arabia and the Gulf region to examine gender-specific associations of plaques and blood lipids. The study examined a large number of patients seen over 10 years in a large referral center. This allowed gender specific presentation of data. Both univariate and multivariate analysis was used to detect independent associations. Nevertheless, few limitations are acknowledged. The cross-sectional design can determine association but not causation. Being a single center experience without follow up data about clinical CAD may limit the generalizability of the findings. However, it should be fine for a similar population without evidence of clinical CAD. As the patients were first seen at the CT, there was lack of information about the use of and adherence with lipid lowering medications. Patient rather than segmental plaque assessment may have overestimated calcified plaques. However, we believe that these limitations have minor impact on the study finding (if any).

In conclusion, middle-aged patients without clinical CAD in Saudi Arabia have a high burden of plaques, specially calcified ones. Plaque-gender interactions were detected in HDL cholesterol and triglycerides. Males who had higher burden of plaques had higher triglycerides and lower HDL cholesterol compared with females. Plaques were associated with lower HDL cholesterol and higher triglycerides, with stronger associations in males than females in both univariate and multivariate analysis. The current findings may impact the use of lipid lowering mediations, by underscoring the importance of assessing the risk of CAD in patients without clinical CAD even in case of lack of coronary calcification.

## Data Availability

The datasets generated and/or analyzed during the current study are not publicly available as per the Ministry of defense and local guidelines of Prince Sultan Cardiac Centre but are available from the corresponding author on reasonable request; Dr Sumaya Al Helali (sumaya_harbi@yahoo.com, 00966530787548).
